# Acknowledgment to the Reviewers of *Epigenomes* in 2022

**DOI:** 10.3390/epigenomes7010003

**Published:** 2023-01-13

**Authors:** Epigenomes Editorial Office

**Affiliations:** MDPI AG, St. Alban-Anlage 66, 4052 Basel, Switzerland

High-quality academic publishing is built on rigorous peer review. *Epigenomes* was able to uphold its high standards for published papers due to the outstanding efforts of our reviewers. Thanks to the efforts of our reviewers in 2022, the median time to first decision was 20.5 days and the median time to publication was 44 days. Regardless of whether the articles they examined were ultimately published, the editors would like to express their appreciation and thank the following reviewers for the time and dedication that they have shown *Epigenomes*:
Agnieszka Münster-WandowskiLai N. ChanAishwarya GurumurthyLili LiAlexey V. FeofanovMaksim ErokhinAline ProbstMark MorrisAnuj AhujaMasanori SomeyaBambarendage PereraMaximiliano M. PortalBelén Gómez-GonzálezMichael LuebbertBenjamin RocheMinoo RassoulzadeganBing YangMirunalini RavichandranBrandon PierceMohammad AlzrigatCarissa M. KraneMuyan ChenCarla CisternasNaveen Kumar C. GowdaChen CaiNelly OlovaChristian Griñán-FerréPatrik Ferreira VianaChristophe TatoutPatryk KrzeminskiChunyuan JinPaul FranszColin KennyPaul WadeEleonora LoiPradeepa MadapuraEric ConwayRichard S. JonesEstanis NavarroRichard S. LeeFanju MengRodrigo MaldonadoFrédéric BantigniesSarah LaMereFumika YakushijiShiyi YinGail A. CornwallSimone BaldanziGeorge ZachosSitaram GayatriGiulia AbateStaffan Holmberg-ThydénGuohong LiSteve JacobsenHans KnechtTakashi UmeharaHuu Phuc NguyenTed Brown


